# Autoimmune pancreatitis masquerading as carcinoma head of pancreas: A case report and review of literature

**DOI:** 10.1016/j.amsu.2019.07.026

**Published:** 2019-07-28

**Authors:** Meenu Gill, Komal Brar, Rajesh Godara, Shilpi Bhargava, Bhawna Sachdeva, Rajeev Sen, Promil Jain

**Affiliations:** a Department of Pathology, Pt. B D Sharma PGIMS, Rohtak, India; b Department of Surgery, Pt. B D Sharma PGIMS, Rohtak, India; c Max Hospital, Rohini, New Delhi, India

**Keywords:** Pancreatitis, Autoimmune, Carcinoma, AIP- Autoimmune Pancreatitis, CT-Computed Tomography

## Abstract

**Introduction:**

Autoimmune pancreatitis (AIP) is a rare form of chronic inflammatory pancreatic disease secondary to an underlying autoimmune mechanism. It is now considered as pancreatic manifestation of IgG4 related disease, which is a multisystem disease.

**Case report:**

We are reporting a patient who presented with obstructive jaundice and mass head of pancreas on Computed Tomography (CT) scan. Considering a strong clinical suspicion of pancreatic cancer, Whipple procedure was done. Histopathological report revealed intense lymphoplasmacytic infiltrate and fibrosis with collagenisation, so possibility of AIP was suggested. Serum IgG4 levels were advised and found to be increased. Diagnosis of AIP was made and patient responded to steroids.

**Discussion:**

Pre-operative core biopsy of the pancreas and Serum IgG4 levels are sufficient to make the diagnosis and resection is usually not recommended in AIP.

**Conclusion:**

Awareness of the entity and use of ancillary techniques in making the pre-operative diagnosis could have saved the patient from an extensive surgical procedure.

## Highlights

• Autoimmune Pancreatitis is a pancreatic manifestation of IgG4 related disorders.
• It can clinically and radiologically present as pancreatic malignancy.
• Resection is not required in Autoimmune Pancreatitis.
• Pre-operative investigations can save the patient from morbidity due to Whipples.
• Whipples procedure is undesired in these patients.
• This entity should be kept in mind while dealing with mass lesions in head of pancreas.

## 1 Introduction

Autoimmune pancreatitis (AIP) is a rare form of chronic inflammatory pancreatic disease secondary to an underlying autoimmune mechanism. Various terms used to describe it are lymphoplasmacytic sclerosing pancreatitis, non-alcoholic duct destructive chronic pancreatitis, and pseudotumor pancreatitis [1]. Now, it is well understood that AIP is of two types, type 1 is considered as pancreatic manifestation of IgG4 related disease, which is a multisystem disease. Type 2 AIP is not related to IgG4 and is a pancreas specific disorder [2]. We are reporting a patient who presented with obstructive jaundice and mass head of pancreas on Computed Tomography scan (CT) and mildly elevated CA19.9 levels. Considering a strong clinical suspicion of pancreatic cancer, Whipple procedure was done. Histopathological report revealed features of AIP and patient was treated effectively with steroids. Use of ancillary techniques would have helped in making the pre-operative diagnosis and saved the patient from an extensive surgical procedure.

### 1.1 Case report

A sixty year old Asian nonalcoholic female presented in Surgery Clinic with chief complaints of pain abdomen off and on since 6–7 months which was non-radiating. Patient also had loss of weight and appetite since 4 months. She also complained of on and off joint pain involving distal joints for last ten years, often relieved by NSAIDS. There were no associated risk factors or diseases like ulcerative colitis. Physical examination showed scleral icterus and abdominal examination revealed slight deep tenderness in epigastrium. Lab investigations revealed CA 19.9–110 U/ml, CEA- 1.5 ng/ml, Total bilirubin- 12.2mg/dl, Direct- 7.6 mg/dl, Indirect- 4.6 mg/dl, Aspartate aminotransferase (AST)-132 IU/L and alanine aminotransferase (ALT)-216 IU/L.

**Fig. 1:**
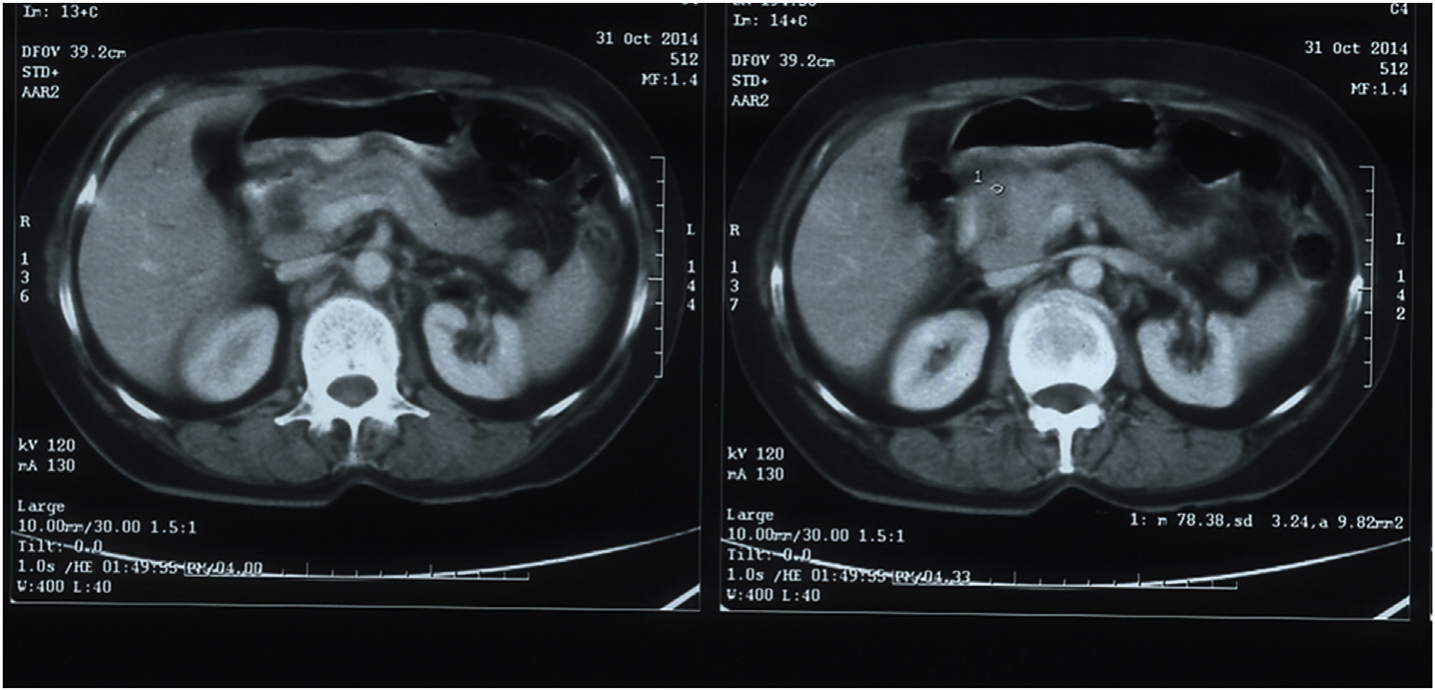
CECT Abdomen revealing bulky pancreatic head with lobulated outline and ill-defined hypodense mass lesion.

Ultrasonography abdomen showed an ill-defined hypoechoic mass lesion in head of pancreas measuring 3.1 cm × 2.7 cm with mildly dilated main pancreatic duct (MPD). CT Abdomen revealed an ill-defined hypodense mass lesion in pancreas causing bilobar IHBR dilatation and mild MPD dilatation with peripancreatic lymphadenopathy (Fig. 1). Few lymph nodes seen at portahepatis, around pancreatic head (7–9mm), and along right common iliac vessels.

Depending upon clinical, biochemical and imaging profile a pre-operative diagnosis of malignancy head of pancreas was considered. Pre-operative tissue diagnosis was not made. Patient underwent explorative laparotomy and Whipple procedure was performed. Specimen sent for histopathological examination (HPE) comprised of part of stomach, duodenum, head of pancreas and part of jejunum. Cut-section of head of pancreas showed a circumscribed grey white, scirrhous area measuring 3.2 cm × 2.5cm × 2.0 cm. Hepatic, choledochal and mesenteric lymph nodes were also received separately.

**Fig. 2:**
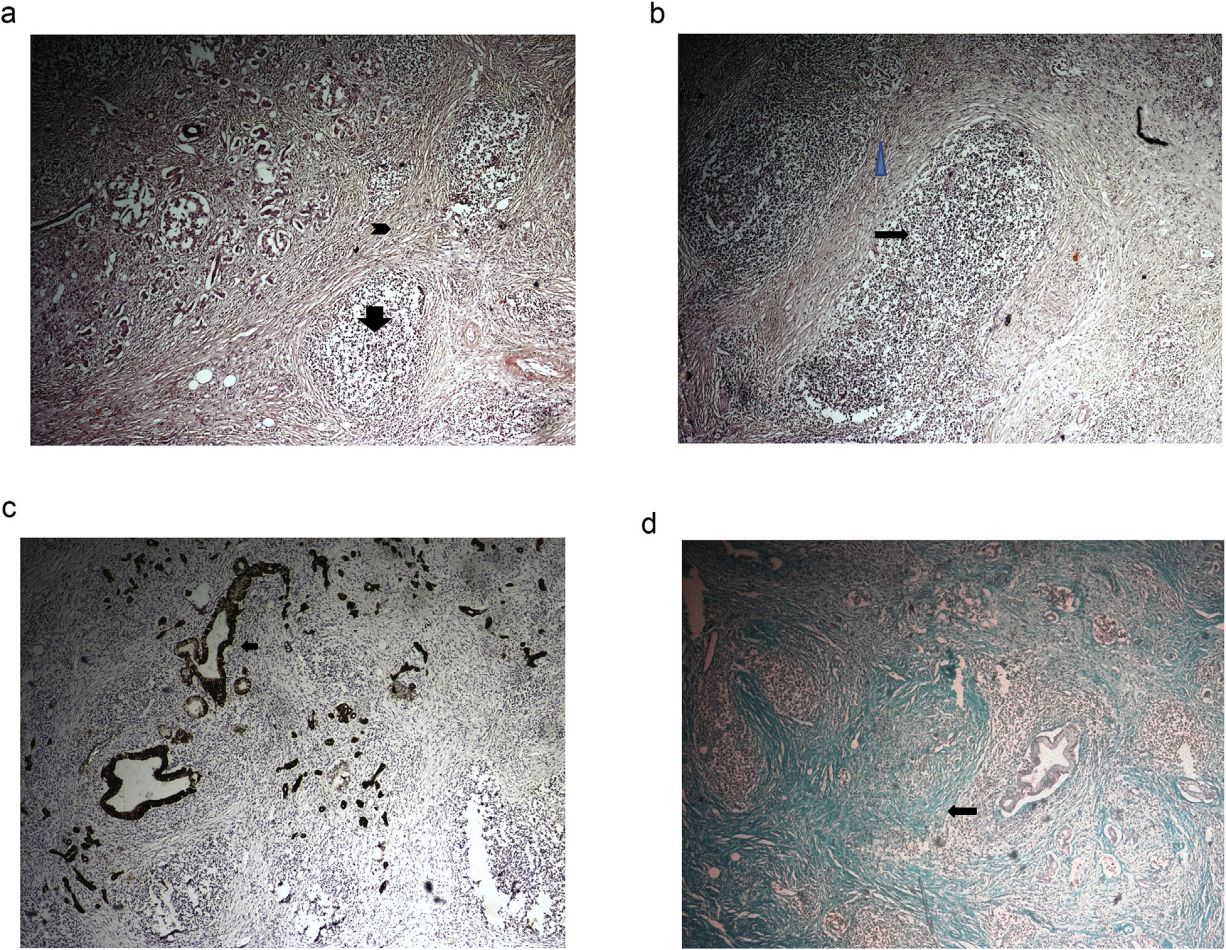
a&b: Dense stromal sclerosis (arrow head) and Lymphoplasmacytic infiltrate with acinar cell destruction (arrow); (H&E; 40x). c: Normal ductules showing cytoplasmic positivity (Immunohistochemistry,CK; 40x). d: Areas of dense fibrosis (Masson's Trichome stain; 40x).

Haematoxylin & eosin stained sections from the grey white firm looking areas revealed intense lymphoplasmacytic infiltrate and fibrosis with collagenisation causing destruction of acinar cells and compression of ductules (Fig. 2a and b). The normal pancreatic parenchyma was nearly replaced by chronic fibroinflammatory reaction. The normal ductules were positive for Cyto-keratin (CK) (Fig. 2c) and Masson's Trichome stain was used to delineate areas of fibrosis (Fig. 2d). Entire specimen including lymph nodes was negative for malignancy. Based on extensive fibroinflammatory reaction resulting in obstruction of biliary ductular system and obstructive jaundice, possibility of autoimmune pancreatitis was suggested and serum IgG4 levels were advised post surgery. S.IgG4 levels were elevated to 334 mg/dl (normal range 11–157 mg/dl). Final diagnosis of Autoimmune pancreatitis was made. Patient was put on prednisone 40 mg/day for 4 weeks, and showed complete improvement, this was followed by gradual tapering of steroids. But unfortunately, after 6 months she was admitted again with complaints of chest pain and breathlessness. She had developed pleural effusion and ascites (owing to polyserositis which is also a complication of autoimmune disorders). Multiple pleural and peritoneal taps had to be performed over a period of next 8–10 days but patient's condition deteriorated and she expired about 20 days after admission due to polyserositis. This case is reported in line with the SCARE criteria [3].

## 2 Discussion

AIP is a rare disease with incidence around 0.82 per 100,000 in Japan. The incidence in western nations and rest of the world is also low. Incidence of AIP has risen dramatically now due to increased radiological and serological diagnostic modalities which can preoperatively help in differentiating the malignant and inflammatory non-neoplastic pathologies [4].

AIP accounts for approximately 27% of Whipple resections done for suspicious pancreatic adenocarcinoma, which is a significant number and cannot be ignored [5]. Many investigations have been explored to diagnose it preoperatively so that number of extensive surgeries can be decreased, since AIP can be treated with steroids [6]. Elevated CA 19-9 levels are characteristic for pancreas adenocarcinoma; and are rarely increased in AIP patients. Endoscopic retrograde cholangiopancreatography (ERCP) images are typical of AIP, and combining with any other positive result can confirm the diagnosis of AIP [7].

In a Korean study including 67 patients of AIP, 12 patients were operated due to a false diagnosis of pancreatic carcinoma [8]. In a study conducted by Hammani et al. a 27-year-old man presented with obstructive jaundice and radiographic image of pancreatic cancer. Whipple operation was performed and histopathological examination revealed AIP. Patient responded to steroids with resolution of disease [9]. Like present study, no preoperative tissue diagnosis was made in the study mentioned above, had it been done, patient could have been saved from morbidity associated with Whipple procedure.

The most common clinical presentation in AIP is painless obstructive jaundice, which can also be confused with pancreatic cancer [10]. Two types of AIP have been described. Type 1 is the more recognized form of the disease. It is the pancreatic manifestation of IgG4 related systemic disease. Lymphoplasmacytic sclerosing pancreatitis (LPSP) is the main histological finding i.e. dense lymphoplasmacytic infiltrate particularly rich in IgG4 positive cells. Storiform fibrosis and obliterative phlebitis are also characteristic features. It is often associated with extrapancreatic lesions. Unlike most of the autoimmune processes, AIP is more common in males, with M: F ratio of 2:1. Type 2 is a recently described form. It consists of an idiopathic duct-centric pancreatitis (IDCP) i.e., inflammation is centered on the exocrine pancreatic system, with neutrophilic infiltration within the lumen and epithelium of the interlobular ducts, sometimes forming microabscesses. Type 2 AIP is seen in younger patients with no gender preponderance [11].

Other IgG4 related extrapancreatic disorders include Mikulicz's syndrome, retroperitoneal fibrosis, inflammatory pseudotumor, hilar lymphadenopathy, Riedel's thyroiditis, and tubulointerstitial nephritis [2,10].

Although, elevated serum IgG4 levels are characteristic of AIP, they can only be used in conjunction with other diagnostic findings for making the diagnosis. Other elevated antibody markers can be rheumatoid factor, antilactoferrin and antinuclear antibodies and anti-plasminogen-binding peptide antibody [11,12].

CT shows a swollen, “sausage-like” pancreas. Borders have a capsule-like low-density rim and diffuse narrowing of the pancreatic duct is due to periductal inflammation. Endoscopic retrograde cholangiopancreatography (ERCP) shows a long, narrow ductal stricture or multiple non-continuous strictures. ERCP is helpful in making the diagnosis of AIP. EUS is also a good investigation useful for this purpose. However, small material obtained from EUS-guided fine-needle aspiration is usually insufficient to make a diagnosis of AIP. A core biopsy of the pancreas is required for definitive diagnosis [10].

Several types of diagnostic criteria have been proposed which aid in diagnosis of this entity. Japanese Group used **I**maging, **S**erology and **H**istology for diagnosing AIP. Mayo clinic has given HISORt criteria for diagnosis of AIP which require **H**istology, **I**maging, **S**erology, **O**ther organ involvement and **R**esponse to **t**herapy (steroids) for diagnosis [13].

International association of pancreatology has devised international consensus on the diagnostic criteria (ICDC). According to it terms type 1 and type 2 should be used to describe the clinical profiles associated with LPSP and idiopathic duct-centric pancreatitis (IDCP), respectively. Only ICDC describes features of type 2 AIP [5]. ICDC 2011 criteria is detailed and most up-to-date knowledge on both types of AIP, but it is too complicated for clinics. Mayo clinic's HISORt criteria can be easily used in daily clinics [11].

The treatment of choice for autoimmune pancreatitis is steroids. After a starting high dose for 4 weeks, gradual tapering can be done, once the symptoms of jaundice regress. Resection is usually not recommended, it is done only in cases misdiagnosed as malignancy. Core biopsy of the pancreas is sufficient to make the diagnosis [13].

## 3 Conclusion

AIP is a unique chronic pancreatic disorder and its presentation can be quite similar to pancreatic adenocarcinoma. Its clinical suspicion is critical in patient's management. As it has an excellent response to corticosteroid therapy, expertise of the doctors in diagnosing this entity can save the patient from extensive surgery and resulting morbidity.

## Ethical approval

No research ethics approval was necessary for this case report. Written informed consent was obtained from the patient for publication of this Case report and any accompanying images. A copy of the written consent is available for review by the Editor-in-Chief of this journal.

## Funding

Nil.

## Author contribution

Please specify the contribution of each author to the paper, e.g. study design, data collections, data analysis, writing. Others, who have contributed in other ways should be listed as contributors.

Study concept: Dr. Meenu Gill, Dr. Rajesh Godara.

Writing the paper: Dr. Komal Brar, Dr. Shilpi Bhargava, Data Interpretation: Dr. Rajeev Sen, Dr. Promil Jain, Dr Bhawna Sachdeva.

## Conflict of interest

We have no conflict of interest.

## Research registration unique identifying number (UIN)

None.

## Guarantor

Dr. Komal Brar.

## Consent

Written informed consent was obtained from the patient for publication of this case report and accompanying images. A copy of the written consent is available for review by the Editor-in-chief of this journal on request.

## Provenance and peer review

Not commissioned, externally peer reviewed.
